# The hnRNPK/A1/R/U Complex Regulates Gene Transcription and Translation and is a Favorable Prognostic Biomarker for Human Colorectal Adenocarcinoma

**DOI:** 10.3389/fonc.2022.845931

**Published:** 2022-07-07

**Authors:** Yixin Li, Hui Wang, Jiajia Wan, Qian Ma, Yu Qi, Zhuoyu Gu

**Affiliations:** ^1^ Department of Clinical Oncology, The First Affiliated Hospital of Zhengzhou University, Zhengzhou University, Zhengzhou, China; ^2^ Post-Doctoral Station of Clinical Medicine, The First Affiliated Hospital of Zhengzhou University, Zhengzhou University, Zhengzhou, China; ^3^ Department of Thoracic Surgery, The First Affiliated Hospital of Zhengzhou University, Zhengzhou University, Zhengzhou, China

**Keywords:** colorectal adenocarcinoma, hnRNPK complex, prognosis, biomarker, transcription, translation

## Abstract

Heterogeneous nuclear ribonucleoproteins (hnRNPs) are emerging as a crucially important protein family in tumors. However, it is unclear which family members are essential for cancer progression, and their diverse expression patterns and prognostic values are rarely reported. In this work, we found that the expression levels of hnRNPs were all upregulated in colon adenocarcinoma (COAD) and rectal adenocarcinoma (READ) tissues. Immunohistochemical staining revealed that hnRNPA1, hnRNPA2B1, hnRNPC, hnRNPK, hnRNPR, and hnRNPU are overexpressed in colorectal adenocarcinoma. Additionally, the promoter methylation levels of hnRNPs were significantly elevated or decreased, and multiple genetic alterations of hnRNPs were found in colorectal adenocarcinoma patients. Correlation analysis showed that the expression levels of hnRNPs were positively correlated with each other. Furthermore, we demonstrated that high expressions of hnRNPA1, hnRNPK, hnRNPR, and hnRNPU were associated with better overall survival rates for colorectal adenocarcinoma patients. The co-expression network and functional prediction analysis indicated that hnRNPK/A1/R/U was involved in cellular gene transcription and translation. Moreover, hnRNPK/A1/R/U complex was identified and confirmed by mass spectrometry and co-immunoprecipitation. RNA sequencing analysis revealed that the transcription factor hnRNPK regulated transcription and translation of related genes. Finally, through establishment of stable cell lines *in vitro*, we verified that hnRNPK was a favorable factor in human colorectal adenocarcinoma which promoted immune cell infiltration and inhibited tumor growth. Our findings illustrate that the hnRNPK/A1/R/U complex is a favorable prognostic biomarker for human colorectal adenocarcinoma. Targeting hnRNPK during transcription and translation could be a promising therapeutic strategy for colorectal adenocarcinoma treatment.

## Introduction

Colorectal cancer (CRC) is one of the most aggressive malignant tumors in the world. In 2018, the morbidity and mortality of CRC were 10.2% and 9.2%, respectively, ranking the third and the second in all human cancers (1). CRC patients in China account for 24.3% of new global CRC cases, with 28% incidence and 13% mortality (2, 3), seriously affecting public health. Colorectal adenocarcinoma is the most common histopathological type of CRC, and mainly includes colon adenocarcinoma (COAD) and rectal adenocarcinoma (READ). Owing to the rapid growth and strong invasiveness of COAD and READ cells, so far, the prognosis of patients with colorectal adenocarcinoma is still poor (4). Currently, few targeting molecules are effective in CRC clinical trials (5). Therefore, the identification of new biomarkers for diagnosis and treatment of colorectal adenocarcinoma is essential and urgent.

Heterogeneous nuclear ribonucleoproteins (hnRNPs) are a family of nucleic acid-binding proteins (6). Recently, the role of hnRNPs in cancer development has attracted attention. In many types of cancers, the expression levels of hnRNPs are significantly altered, suggesting its crucial role in tumorigenesis. It has been reported that hnRNPs are involved in multiple tumor-associated events, such as regulating cell survival, migration, metabolism, and stress response (7, 8). However, the role of hnRNPs in digestive system tumors, especially in colorectal adenocarcinoma, has not been fully elucidated. So far, the mechanism of action and clinical value of hnRNPs in colorectal adenocarcinoma remain unclear, but are important to understand for diagnosis and targeted therapy of colorectal adenocarcinoma.

Currently, eight hnRNPs members (hnRNPA1, hnRNPA2B1, hnRNPC, hnRNPD, hnRNPF, hnRNPK, hnRNPR, and hnRNPU) seem to be more relevant to cancer development, according to reported literature (6–8). In detail, hnRNPA1 is a multifunctional RNA binding protein which is involved in pre-mRNA splicing, export of mature transcripts from the nucleus, mRNA turnover, and internal ribosome entry site (IRES)-mediated translation. Recently, hnRNPA1 is indicated as the oncogene in the pathogenesis of hepatocellular cancer and colon cancer, and hnRNPA1 promotes cell proliferation by regulating translation of diverse antiapoptotic proteins (9–11). HnRNPK is located in the nucleoplasm and has three repeats of KH domains that binds to RNAs. This protein is a conserved pre-mRNA-binding protein that is involved in multiple processes of gene expression, including chromatin remodeling, transcription, and mRNA splicing, translation, and stability. HnRNPK is also identified as an oncogene and is associated with prognostic survival in various types of malignancies, but recently hnRNPK is identified as a tumor suppressor in hematologic malignancies, due to its regulation of the p53/p21 signaling pathway activation (12). HnRNPR is a member of the spliceosome C complex, which functions in pre-mRNA processing and transport. During the development of gastric cancer, hnRNPR overexpression can promote the cancer cell survival and metastasis (13). HnRNPU is a component of ribonucleosomes localized in cytoplasmic mRNP granules containing untranslated mRNAs. Similarly, high hnRNPU levels are also observed in gastric cancer tissues and predict poor prognosis in gastric cancer patients, suggesting that hnRNPR and hnRNPU may be tumor-promoting factors in gastric cancer cells (14). Generally, the eight hnRNPs may function as oncogenes or tumor suppressors in tumorigenesis. However, the underlying mechanisms by which hnRNPs are activated or inhibited are unclear, and the specific functions of the eight hnRNPs are still unknown. Importantly, it is unclear which family members are essential for cancer progression, and their diverse expression patterns and prognostic values are rarely reported.

To date, bioinformatics has not been used to investigate the role of hnRNPs in colorectal adenocarcinoma. In this work, to obtain expression patterns, functions, and prognostic values of hnRNPs in colorectal adenocarcinoma. A complex bioinformatics analysis through online webtools was performed. Additionally, immunohistochemistry (IHC), co-immunoprecipitation (Co-IP), western blot (WB), RNA sequencing (RNA-seq), IncuCyte system, and other *in vitro* experiments were used to verify the diverse expression patterns and prognostic values. The aim of this work was to provide promising therapeutic targets for colorectal adenocarcinoma treatment.

## Materials and methods

### Cell Culture and Construction of Stable Cell Lines

The human colorectal adenocarcinoma cell lines (HCT116, SW480, and SW620) and human normal colonic epithelial cells (FHC) were obtained and authenticated from Cell Bank of Chinese Academy of Sciences (Shanghai, China) and cultured in RPMI-1640 medium supplemented with 10% fetal bovine serum. All cells were maintained in an incubator with a humidified atmosphere of 5% CO_2_. The lentiviruses of hnRNPK knockdown (cat# GIDL0235518, sh1: 5´-3´AGATTTGGCTGGATCTATT, sh2: 5´-3´CGTTATTGTTGGTGGTTTA) and hnRNPK overexpression (cat# GCD0235440) were purchased from Shanghai GeneChem Co. (Shanghai, China). Negative control shRNA cells (shNC: 5´-3´TTCTCCGAACGTGTCACGT) and empty vector-infected cells (vector) were established as controls. Stable cell lines were established *via* selection with 1 μg/mL puromycin for 4 weeks.

### Silver Staining and Mass Spectroscopy (MS)

The protein gel was immersed in stationary liquid with 10% acetic acid, 50% ethanol and 40% water at room temperature on a shaker overnight, and the protein bands were subsequently visualized using the Protein Fast Silver Stain Kit from Beyotime Co. (Shanghai, China) according to the manufacturer’s instruction, and binding proteins were analyzed *via* mass spectrometry (MS) from Wininnovate Co. (Shenzhen, China).

### Co-IP and WB

Nucleoprotein extracts were treated with RNase (Solabio, China) and incubated with antibodies for hnRNPK (1:50) or IgG for 24 h on a rotating wheel. Thereafter, the Sepharose-conjugated protein-A/G beads were added and incubated at 4°C for another 24 h on a rotating wheel. After extensive rinsing with cold PBS, the beads were boiled, and the precipitated proteins were separated *via* SDS-PAGE and transferred onto PVDF membranes. The membranes were blocked with 5% non-fat milk for 2 h at room temperature and subsequently incubated with primary and secondary antibodies, and protein bands were detected *via* enhanced chemiluminescence. The primary antibodies used were as follows: hnRNPK (Proteintech, #11426-1-AP, 1:1000), IgG (CST, #2729, 1:1000), hnRNPA1 (Proteintech, #11176-1-AP, 1:5000), hnRNPR (Proteintech, #15018-1-AP, 1:1000), hnRNPU (Proteintech, #16365-1-AP, 1:5000), Histone H3 (Abclonal, #A2348, 1:1000) and β-Actin (Proteintech, #20536-1-AP, 1:2000).

### Cell Proliferation and Colony Formation

For cell proliferation, cells were cultured in 96-well plates and placed in the IncuCyte live-cell imaging system (Essen BioScience, USA). Cells were observed using phase microscopy (NiKon, #MRH00101) and calculated by IncuCyte analysis software. For colony formation, cells were seeded at a density of 500 cells per well in 6-well plates and incubated. Two weeks later, cells were fixed with 4% paraformaldehyde and stained by crystal violet. The images of the colonies were captured, and the numbers of the colonies were counted by the software Image-Pro Plus 6.0.

### RNA-seq

*In vitro*, stable hnRNPK-knockdown (5´-3´AGATTTGGCTGGATCTATT) cell lines (HCT116 and FHC) were established. Negative control cells (shNC: 5´-3´TTCTCCGAACGTGTCACGT) were established as controls. Total cellular RNA was extracted for quality control and obtaining RNA-seq loading samples. RNA-seq was performed as DGE on an Illumia HiSeq platform and 50 bp paired-end reads were generated by BGI Co. (Shenzhen, China). The deep sequencing data and processed data were submitted to the NCBI Gene Expression Omnibus (GEO) data repository under the accession number GSE186371 (HCT116 shNC cells and HCT116 shhnRNPK cells) and GSE198445 (FHC shNC cells and FHC shhnRNPK cells).

### Immunohistochemical (IHC) staining

Fresh human tumor tissues and adjacent normal tissues were obtained from 10 patients diagnosed with colorectal adenocarcinoma between April 2020 and June 2021 at the First Affiliated Hospital of Zhengzhou University. Written informed consent was obtained from each patient involved in the study, and the study was approved by the ethics committee of the First Affiliated Hospital of Zhengzhou University. The paraffin-embedded samples were prepared at 4 μm thickness. All the slides were through deparaffinization and rehydration. The slides were then incubated with primary antibodies overnight. The primary antibodies used were as follows: hnRNPK (Proteintech, #11426-1-AP, 1:100), hnRNPA1 (Proteintech, #11176-1-AP, 1:50), hnRNPR (Proteintech, #15018-1-AP, 1:100), hnRNPU (Proteintech, #16365-1-AP, 1:50), hnRNPA2B1 (Proteintech, #14813-1-AP, 1:100), hnRNPD (Proteintech, #12770-1-AP, 1:150), hnRNPF (Proteintech, #14974-1-AP, 1:150) and hnRNPC (Proteintech, #11760-1-AP, 1:100). Finally, HRP-labeled secondary antibody (Santa Cruz, CA, USA) was incubated for 1 h and immunodetection was performed using diaminobenzidine on the next day. The sections were rehydrated with xylene and ethanol, mounted in mounting resin, and examined with a microscope (Olympus, Japan). Two independent pathologists blinded to clinicopathological outcome calculated immunostaining reactions by multiplying the intensity and proportion of positive tumor cells. Briefly, the percentage (0-100%) of stained tumor cells was multiplied by the intensity (1, no or weak staining; 2, moderate staining; 3, strong staining; and 4, very strong staining) to achieve a score between 0 and 400. All the cases were divided into low expression and high expression subjected to the total score (median).

### ONCOMINE Analysis

The transcript level of hnRNPs in diverse types of colorectal cancer tissues was ascertained by the ONCOMINE webtool (https://www.oncomine.org/), with a threshold set as such that *P* < 1E-04, fold change >2, top gene rank 10% (15). The mRNA levels of hnRNPs in cancer tissues were compared with that in normal tissues. Two one-sided *t*-tests were used to evaluate the differences. The cutoff of *P* value was defined as 0.01, and the cutoff of fold change value was identified as 1.

### UALCAN and GEPIA analysis

The UALCAN (http://ualcan.path.uab.edu) webtool of TCGA gene expression was used to analyze the promoter DNA methylation levels of hnRNPs in colorectal adenocarcinoma tissues and normal tissues (16). The methylation levels of hnRNPs in cancer tissues were compared with that in normal tissues. Two one-sided *t*-tests were used to evaluate the differences. The GEPIA (http://gepia.cancer-pku.cn) webtool of TCGA gene expression was used to analyze the expression of hnRNPs in tumor tissues and normal tissues (17). Two one-sided *t*-tests were used to evaluate the differences. The overall survival (OS) of patients was also assessed in GEPIA by Kaplan-Meier survival plot, using a log-rank test to evaluate the differences.

### CBioPortal Analysis

CBioPortal webtool of 382 colorectal adenocarcinoma patients (TCGA, Firehouse Legacy) was used to further visualize and analyze multidimensional cancer genomics data of the eight hnRNPs (www.cbioportal.org) (18, 19). Based on TCGA database, genetic alterations and correlations of the hnRNPs was obtained from cBioPortal. mRNA expression z scores (RNA Seq V2 RSEM) were obtained using a z score threshold of ±2.0. Protein expression z scores (RPPA) were obtained using a z score threshold of ±2.0. The analysis methods were according to the cBioPortal’s online instruction.

### Human Protein Atlas (HPA) analysis

The eight hnRNPs were analyzed at the protein and RNA levels in the HPA webtool (https://www.proteinatlas.org/), which contained IHC stained images of 597 clinical colorectal adenocarcinoma patients and their survival information with 275 females and 322 males (20, 21). The OS of patients with colorectal adenocarcinoma was assessed by a Kaplan-Meier survival plot, using a log-rank test to evaluate the differences.

### Coexpedia and DAVID analysis

Coexpedia webtool was used to establish the co-expression networks of the genes in mammals (http://www.coexpedia.org/) (22). To investigate the interaction between genes associated with hnRNPK/A1/R/U, a co-expression network related to hnRNPK/A1/R/U was constructed. The visualization of the network was ranked according to Coexpedia’s online instructions. DAVID 6.8 (https://david.ncifcrf.gov/home.jsp) is a comprehensive, functional annotation website that helps investigators better clarify the biological function of submitted genes. The functional prediction of hnRNPK/A1/R/U was evaluated by Gene Ontology (GO) term analysis and Kyoto Encyclopedia of Genes and Genomes (KEGG) pathway analysis in the DAVID webtool (23, 24). In our study, the correlated genes (top99) of hnRNPK/A1/R/U were directly obtained from CBioPortal webtool, the analysis was visualized with R project using a “ggplot2” package and *P* < 0.05 was set as the cut-off criterion. Biological processes (BP), cellular components (CC), and molecular function (MF) were included in the GO enrichment analysis. All the analyses were performed according to DAVID’s online instructions.

### TIMER analysis

The Tumor Immune Estimation Resource (TIMER2.0) (http://timer.cistrome.org/) is a public webtool for comprehensive assessments of abundance of tumor-infiltrating immune cells (TIICs) according to gene expression profiles (25). We mainly performed hnRNPK expression in tumors and the correlation between gene expression and abundance of immune infiltrates, involving B cells, CD8+ T cells, and CD4+ T cells, macrophages, neutrophils, dendritic cells (DCs), compared with purity in tumors.

### Statistical analysis

Statistical analyses were performed by SPSS 22.0 software, and the results were presented as the mean ± standard deviation (SD). Student’s *t*-test was performed to detect significant differences between groups. Analyses with more than two groups were performed by the one-way ANOVA test. The correlation was assessed using Spearman rank correlation analysis, and OS curves were assessed using Kaplan-Meier analysis. *P* < 0.05 was considered statistically significant.

## Results

### The expression levels of hnRNPs were upregulated in colorectal adenocarcinoma tissues

**Figure 1:**
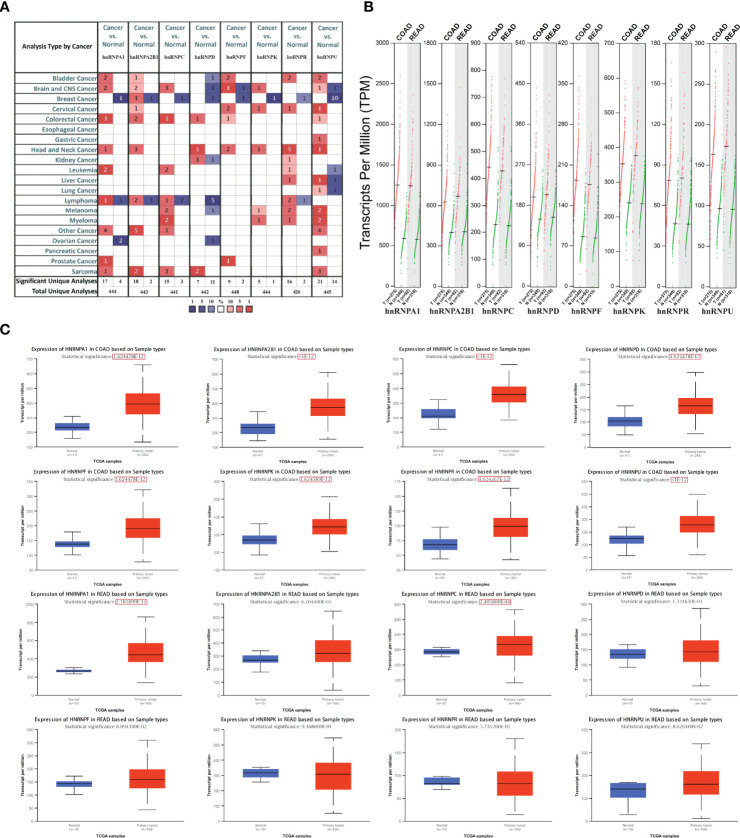
The expression levels of hnRNPs were upregulated in colorectal adenocarcinoma tissues. **(A)** The transcriptional levels of hnRNPs were observed in cancer samples by using ONCOMINE databases. As shown, the expression levels of hnRNPs were upregulated in colorectal cancer tissues compared with the normal tissues. **(B)** In GEPIA database, the transcripts per million (TPM) levels of hnRNPA1/A2B1/C/D/F/K/R/U were upregulated in COAD and READ tissues. **(C)** In UALCAN databases, the TPM levels of hnRNPA1/A2B1/C/D/F/K/R/U were upregulated in COAD tissues compared with the normal tissues. In READ tissues, the TPM levels of hnRNPA1/C were statistically upregulated, and hnRNPA2B1/D/F/K/R/U exhibited the upregulated trends.

**Figure 2:**
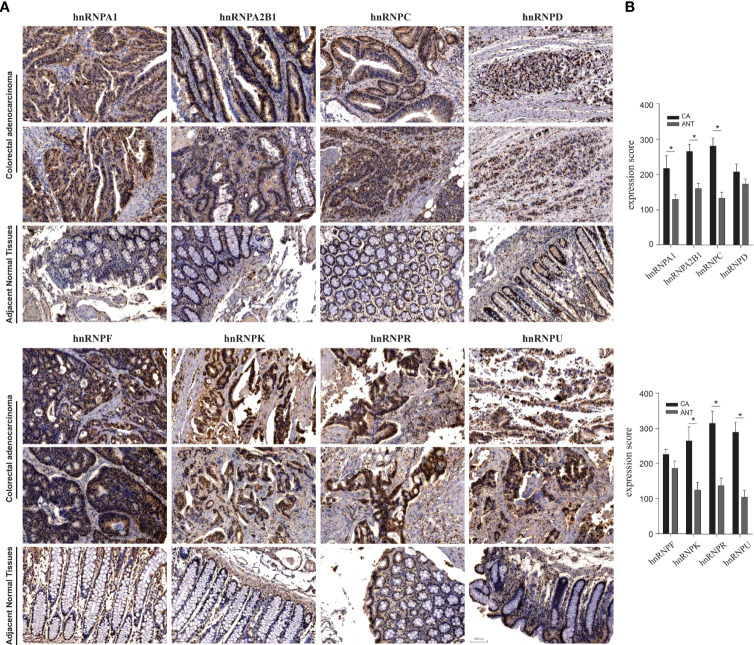
IHC staining of hnRNPs family members in 10 human colorectal adenocarcinoma specimens. By using IHC staining of 10 human colorectal adenocarcinoma specimens, we confirmed that hnRNPA1, hnRNPA2B1, hnRNPC, hnRNPK, hnRNPR, and hnRNPU were mainly localized in the nucleus and had higher expressions in colorectal adenocarcinoma tissues compared with adjacent normal tissues (ANT). *P < 0.05.

We first observed the transcriptional levels of hnRNPs in cancer samples by using ONCOMINE webtool (**Figure 1A** and **Table 1**). As shown, the expression levels of hnRNPs were upregulated in CRC tissues compared with the normal tissues. In the ONCOMINE, overexpression of hnRNPA1 was found in colorectal adenocarcinoma compared with normal samples as follows: COAD with fold change = 2.424, rectal adenoma with fold change = 2.120 and rectal mucinous adenocarcinoma with fold change = 1.620 (26–28). Similarly, high hnRNPK levels were also observed in colorectal adenocarcinoma compared with normal samples (COAD with fold change = 1.574, colorectal carcinoma with fold change = 1.462, and rectosigmoid adenocarcinoma with fold change = 1.215) (26, 28, 32). In Kaiser’s dataset (28), hnRNPR was overexpressed in the colorectal adenocarcinoma types compared with that in the normal samples (COAD with fold change = 1.386, rectosigmoid adenocarcinoma with fold change = 1.688, and cecum adenocarcinoma with fold change = 1.436). Another increased mRNA expression factor was hnRNPU with a fold change = 1.657 in patients with COAD, fold change = 1.900 in patients with colon carcinoma, and fold change = 2.151 in patients with rectal adenoma (27, 29, 34). For hnRNPA2B1/C/D/F, a similar trend was found. The levels of hnRNPA2B1/C/D/F were upregulated in colorectal adenocarcinoma tissues with certain fold changes (27–34). Furthermore, through GEPIA, UALCAN, and TIMER webtools, we revealed that the transcripts per million (TPM) levels of hnRNPA1/A2B1/C/D/F/K/R/U were upregulated in COAD and READ tissues compared with normal tissues (**Figures 1B, C**). By using IHC staining of 10 human colorectal adenocarcinoma specimens, we confirmed that hnRNPA1, hnRNPA2B1, hnRNPC, hnRNPK, hnRNPR, and hnRNPU were mainly localized in the nucleus and had higher expressions in colorectal adenocarcinoma tissues compared with adjacent normal tissues (ANT) (**Figures 2A, B**).

**Table 1:** The changes of hnRNPs expression between diverse types of colorectal cancer and colorectal tissues (ONCOMINE webtool).

	Types of colorectal cancer vs. Normal	Fold change	*P* value	*t*-test	Ref
hnRNPA1	Colon Adenocarcinoma vs. Normal	2.424	3.47E-08	6.497	Alon (26)
	Rectal Adenoma vs. Normal	2.120	8.92E-06	8.530	Sabates-Bellver (27)
	Rectal Mucinous Adenocarcinoma vs. Normal	1.620	2.93E-04	6.438	Kaiser (28)
hnRNPA2B1	Colon Carcinoma vs. Normal	4.242	5.98E-09	14.508	Skrzypczak (29)
	Rectal Adenocarcinoma vs. Normal	1.157	1.35E-07	6.242	Kurashina (30)
hnRNPC	Colon Adenocarcinoma vs. Normal	1.769	1.01E-07	5.737	Ki (31)
	Rectal Adenoma vs. Normal	2.079	1.22E-11	13.006	Sabates-Bellver (27)
	Rectal Mucinous Adenocarcinoma vs. Normal	2.147	3.22E-04	6.707	Kaiser (28)
hnRNPD	Colon Carcinoma vs. Normal	2.033	3.65E-08	10.841	Skrzypczak (29)
	Colorectal Carcinoma vs. Normal	1.813	6.34E-08	8.049	Hong (32)
	Rectal Adenocarcinoma vs. Normal	1.181	6.99E-04	3.269	Gaedcke (33)
hnRNPF	Colon Adenocarcinoma vs. Normal	1.802	3.71E-07	6.226	Notterman (34)
	Rectal Adenoma vs. Normal	2.142	1.06E-05	8.500	Sabates-Bellver (27)
hnRNPK	Colon Adenocarcinoma vs. Normal	1.574	4.36E-04	3.570	Alon (26)
	Colorectal Carcinoma vs. Normal	1.462	1.54E-04	4.557	Hong (32)
	Rectosigmoid Adenocarcinoma vs. Normal	1.215	1.00E-03	3.694	Kaiser (28)
hnRNPR	Colon Adenocarcinoma vs. Normal	1.386	4.91E-04	4.903	Kaiser (28)
	Rectosigmoid Adenocarcinoma vs. Normal	1.688	1.17E-04	6.930	Kaiser (28)
	Cecum Adenocarcinoma vs. Normal	1.436	2.29E-04	4.637	Kaiser (28)
hnRNPU	Colon Carcinoma vs. Normal	1.900	1.80E-06	7.662	Skrzypczak (29)
	Colon Adenocarcinoma vs. Normal	1.657	1.00E-03	3.366	Notterman (34)
	Rectal Adenoma vs. Normal	2.151	3.41E-05	7.377	Sabates-Bellver (27)

### Promoter methylation level, genetic alteration and correlation analysis of hnRNPs were evaluated in human colorectal adenocarcinoma

**Figure 3:**
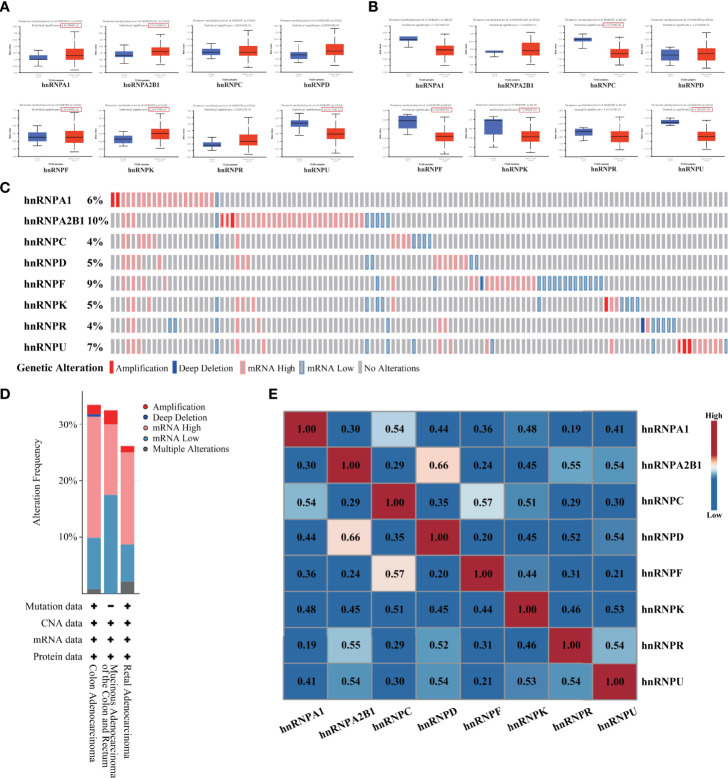
Promoter methylation level, genetic alteration and correlation analysis of hnRNPs were evaluated in human colorectal adenocarcinoma. **(A, B)** In UALCAN database, we found that hnRNPA1, hnRNPA2B1, hnRNPF, and hnRNPK had higher promoter methylation levels in COAD tissues, while hnRNPU had lower promoter methylation levels. In READ tissues, hnRNPC, hnRNPK, hnRNPF, and hnRNPU had lower promoter methylation levels. **(C)** The hnRNPs mutations and correlations were analyzed by using the cBioPortal database. The data showed that hnRNPs were altered in 119 samples out of 382 colorectal adenocarcinoma patients (31%). Two or more alterations were found in almost a third of the samples (36 samples). **(D)** The distribution patterns of the alteration frequency of hnRNPs in colorectal adenocarcinoma were presented. Specifically, the predominant form of genetic alteration occurred in COAD and READ was mRNA upregulation, followed by mRNA downregulation, amplification, and deep deletion. **(E)** The correlations of the eight hnRNPs mRNA levels were analyzed. Pearson’s correlation coefficients revealed that the mRNA levels of hnRNPs with each other were positively correlated.

As demonstrated above, the expression levels of hnRNPs were upregulated in colorectal adenocarcinoma tissues. We further evaluated the promoter DNA methylation levels in colorectal adenocarcinoma tissues. In the UALCAN webtool, we found that hnRNPA1, hnRNPA2B1, hnRNPF, and hnRNPK had higher promoter methylation levels in COAD tissues, while hnRNPU had lower promoter methylation levels. In READ tissues, hnRNPC, hnRNPK, hnRNPF, and hnRNPU had lower promoter methylation levels (**Figures 3A, B**). We then analyzed the hnRNPs mutations and correlations by using the cBioPortal webtool. The data showed that hnRNPs were altered in 119 samples out of 382 colorectal adenocarcinoma patients (31%). Two or more alterations were found in almost a third of the samples (36 samples) (**Figure 3C**). The distribution patterns of the alteration frequency of hnRNPs in colorectal adenocarcinoma were presented. Specifically, the predominant form of genetic alteration occurred in COAD and READ was mRNA upregulation, followed by mRNA downregulation, amplification, and deep deletion (**Figure 3D**). In addition, the correlations of the eight hnRNPs mRNA levels were also analyzed. Pearson’s correlation coefficients revealed that the mRNA levels of hnRNPs with each other were positively correlated (**Figure 3E**).

### High expressions levels of hnRNPA1, hnRNPK, hnRNPR, and hnRNPU were significantly associated with better OS rates in colorectal adenocarcinoma patients

**Figure 4:**
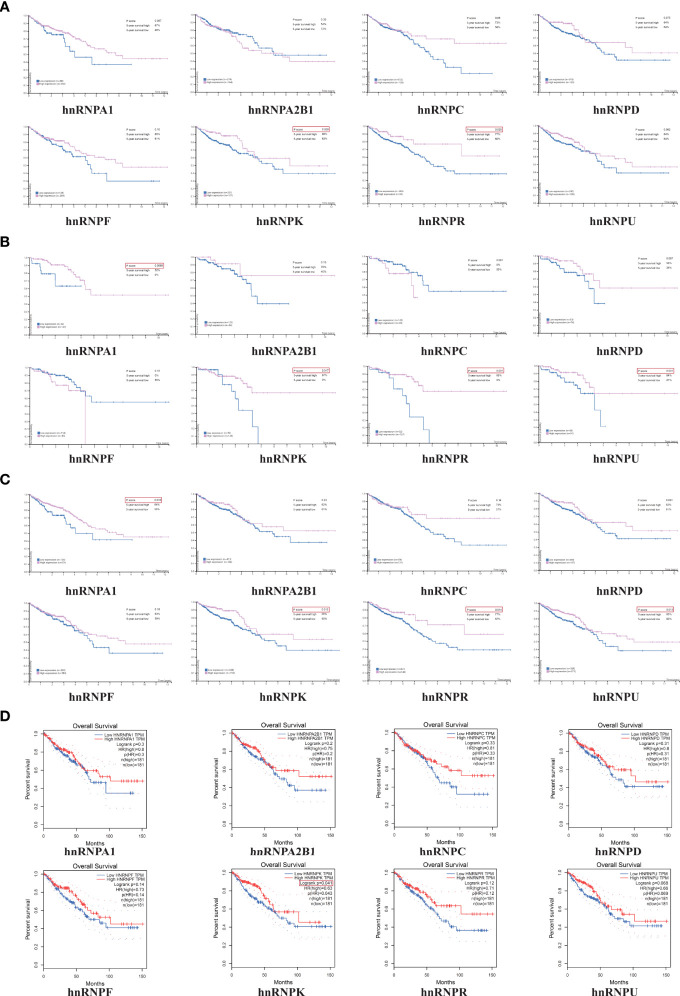
High expressions levels of hnRNPA1, hnRNPK, hnRNPR, and hnRNPU were significantly associated with better OS rates in colorectal adenocarcinoma patients. **(A)** The HPA database was used to examine the relationship between the expression levels of hnRNPs and the survival rates of 597 patients with colorectal adenocarcinoma. The Kaplan-Meier survival analysis demonstrated that high expressions of hnRNPK and hnRNPR were significantly associated with better OS rates for COAD patients. **(B)** High expressions of hnRNPA1, hnRNPK, hnRNPR, and hnRNPU were significantly associated with better OS rates for READ patients. **(C)** High expressions of hnRNPA1, hnRNPK, hnRNPR, and hnRNPU were significantly associated with better OS rates for colorectal adenocarcinoma patients. **(D)** The GEPIA database was selected to further examine the survival of 181 COAD patients and 181 READ patients. Similarly, high expression of hnRNPK was associated with better OS rates.

The HPA webtool was used to examine the relationship between the expression levels of hnRNPs and the survival rates of 597 patients with colorectal adenocarcinoma. The Kaplan-Meier survival analysis demonstrated that high expressions of hnRNPK and hnRNPR were significantly associated with better OS rates for COAD patients (**Figure 4A**). Moreover, high expressions of hnRNPA1, hnRNPK, hnRNPR, and hnRNPU were significantly associated with better OS rates for READ patients (**Figure 4B**). Collectively, high expressions of hnRNPA1, hnRNPK, hnRNPR, and hnRNPU were significantly associated with better OS rates for colorectal adenocarcinoma patients (**Figure 4C**). To confirm the reliability of the results, another independent GEPIA webtool was selected to further examine the survival of 181 COAD patients and 181 READ patients. Similarly, high expression of hnRNPK was associated with better OS rates. Although there were no statistical significances, high expression of hnRNPA1, hnRNPR, and hnRNPU also exhibited the same trends (**Figure 4D**).

### The hnRNPK/A1/R/U network in colorectal adenocarcinoma modulated transcription and translation

**Figure 5:**
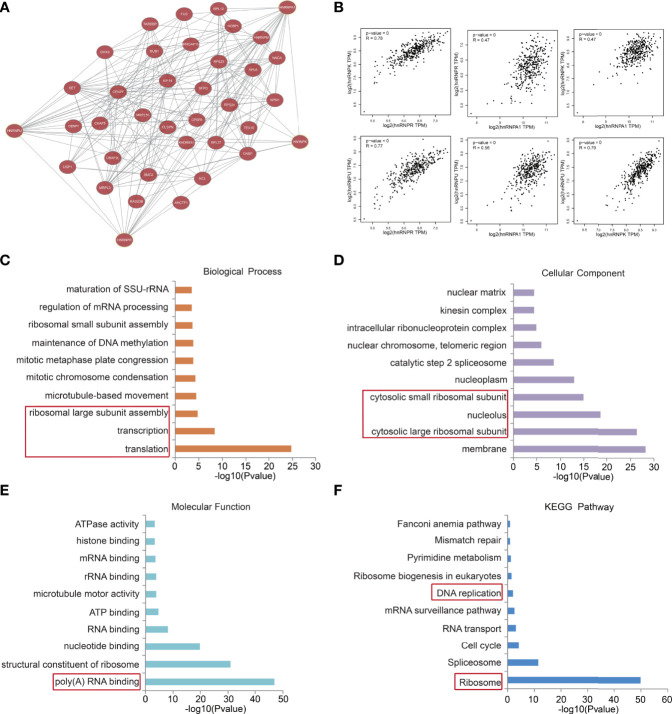
The hnRNPK/A1/R/U network in colorectal adenocarcinoma modulated transcription and translation. **(A)** A gene co-expression network for hnRNPA1, hnRNPK, hnRNPR, and hnRNPU was constructed. The network displayed that the transcription-related and translation-related genes, such as DHX9, MRPL3, SFPQ, CPSF6, RAD23b, and NCBP1, were closely associated with the hnRNPK/A1/R/U network. **(B)** The GEPIA database revealed that the TPM levels of hnRNPK/A1/R/U with each other were positively correlated. **(C, D, E, F)** The functional predictions of hnRNPK/A1/R/U network were analyzed in the DAVID database. The GO analysis results showed that transcription, translation, and ribosomal large subunit assembly were regulated by the hnRNPK/A1/R/U. Furthermore, ribosomal subunit, nucleotide binding, structure constituent of ribosome, and poly(A) RNA-binding were also enriched. The KEGG analysis results showed that DNA replication and ribosomes were enriched, suggesting that hnRNPK/A1/R/U modulated the process of transcription and translation.

We then constructed a gene co-expression network for hnRNPA1, hnRNPK, hnRNPR, and hnRNPU. The network displayed that the transcription-related and translation-related genes, such as DHX9, MRPL3, SFPQ, CPSF6, RAD23b, and NCBP1, were closely associated with the hnRNPK/A1/R/U network (**Figure 5A**). Additionally, the GEPIA webtool revealed that the TPM levels of hnRNPK/A1/R/U with each other were positively correlated (**Figure 5B**). The functional predictions of hnRNPK/A1/R/U network were analyzed in the DAVID webtool. The GO analysis results showed that transcription, translation, and ribosomal large subunit assembly were significantly regulated by the hnRNPK/A1/R/U alterations in colorectal adenocarcinoma (**Figure 5C**). Furthermore, ribosomal subunit, nucleotide binding, structure constituent of ribosome, and poly(A) RNA-binding were also significantly controlled by the hnRNPK/A1/R/U alterations (**Figures 5D** **, E**), and all of these components are well-known processes associated with cell transcription and translation. Finally, through KEGG analysis, ten pathways related to the hnRNPK/A1/R/U network in colorectal adenocarcinoma were identified (**Figure 5F**). More importantly, DNA replication and ribosomes were enriched among these pathways, suggesting that hnRNPK/A1/R/U modulated the process of transcription and translation.

### HnRNPK/A1/R/U complex was identified and validated in human colorectal adenocarcinoma cell lines

**Figure 6:**
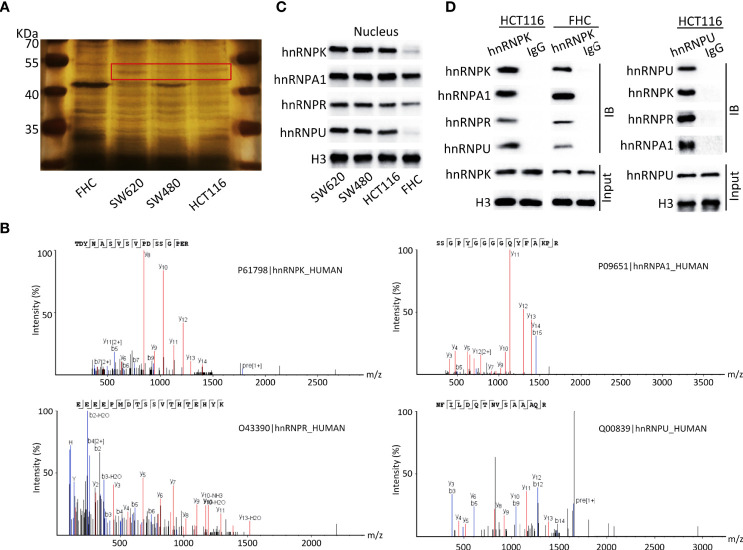
HnRNPK/A1/R/U complex was identified and validated in human colorectal adenocarcinoma cell lines. **(A)** Nuclear proteins of HCT116, SW480, and SW620 cells were extracted, with FHC as the control. After SDS-PAGE and silver staining, a differential protein band with a molecular weight of 50-55 kDa was observed (red rectangle). In detail, more enrichment was observed in SW620 cells and less enrichment was observed in SW480 and HCT116 cells. Importantly, the total enrichment amount of colorectal adenocarcinoma cells was elevated compared with normal cells. **(B)** The protein band in red rectangle was excised, trypsinized, and analyzed by MS. With the best peptide-spectrum sequences, hnRNPA1 (P09651), hnRNPK (P61798), hnRNPR (O43390), and hnRNPU (Q00839) were identified. **(C)** WB assays were performed to validate the expressions of hnRNPA1, hnRNPK, hnRNPR, and hnRNPU in the nucleus, with histone H3 as the control. The results showed that hnRNPA1, hnRNPK, hnRNPR, and hnRNPU in the nucleus had higher expressions in colorectal adenocarcinoma cells compared with FHC cells. **(D)** The interaction between hnRNPK and hnRNPA1/R/U proteins was confirmed by Co-IP in HCT116 cells. To confirm the reliability of this result, the interaction between hnRNPU and hnRNPA1/R/K proteins was further confirmed in HCT116 cells. In FHC cells, the interaction between hnRNPK and hnRNPA1/R/U proteins was observed, which revealed that the hnRNPK/A1/R/U interaction was not specific to cancer cells.

To identify whether hnRNPK/A1/R/U was existed in human colorectal adenocarcinoma, nuclear proteins of HCT116, SW480, and SW620 cells were extracted, with FHC as the control. After SDS-PAGE and silver staining, a differential protein band with a molecular weight of 50-55 kDa was observed (red rectangle). In detail, more enrichment was observed in SW620 cells and less enrichment was observed in SW480 and HCT116 cells. Importantly, the total enrichment amount of colorectal adenocarcinoma cells (HCT116, SW480, and SW620) was elevated compared with normal cells (**Figure 6A**). Subsequently, the protein band in red rectangle was excised, trypsinized, and analyzed by MS. With the best peptide-spectrum sequences in the mass spectra (**Figure 6B**), hnRNPA1 (P09651), hnRNPK (P61798), hnRNPR (O43390), and hnRNPU (Q00839) were identified. Furthermore, WB assays were performed to validate the expressions of hnRNPA1, hnRNPK, hnRNPR, and hnRNPU in the nucleus, with histone H3 as the control. The results showed that hnRNPA1, hnRNPK, hnRNPR, and hnRNPU in the nucleus had higher expressions in colorectal adenocarcinoma cells compared with FHC cells (**Figure 6C**). The interaction between hnRNPK and hnRNPA1/R/U proteins was confirmed by Co-IP in HCT116 cells, ascertaining the existence of hnRNPK/A1/R/U complex in colorectal adenocarcinoma cells. To confirm the reliability of this result, the interaction between hnRNPU and hnRNPA1/R/K proteins was further confirmed in HCT116 cells. In FHC cells, the interaction between hnRNPK and hnRNPA1/R/U proteins was observed, which revealed that the hnRNPK/A1/R/U interaction was not specific to cancer cells (**Figure 6D**).

### RNA-seq in HCT116 cell lines revealed that hnRNPK from hnRNPK/A1/R/U complex mainly regulated cellular transcription and translation

**Figure 7:**
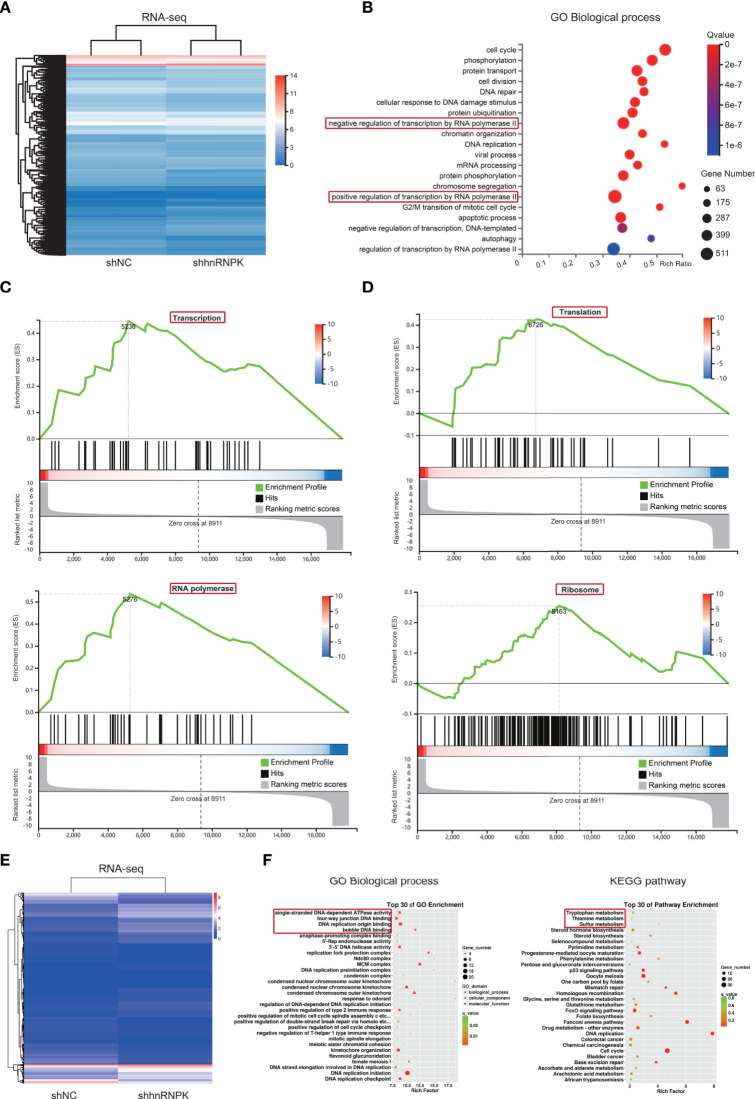
RNA-seq in HCT116 cell lines revealed that hnRNPK from hnRNPK/A1/R/U complex mainly regulated cellular transcription and translation. **(A)** To identify the biological functions of hnRNPK in colorectal adenocarcinoma cells, the primary biological processes that hnRNPK is involved in were analyzed by RNA-seq in HCT116 cells. The heatmap was constructed after hnRNPK knockdown. **(B)** By GO analysis of the differential genes, regulation of transcription by RNA polymerase II was identified as the primary biological function of hnRNPK. **(C, D)** Regulation of transcription by RNA polymerase II and translation by ribosome were identified as the primary biological processes according to the GSEA. **(E, F)** In FHC cells, the primary biological functions of hnRNPK were further analyzed by RNA-seq. By GO analysis of the differential genes, regulation of DNA binding was identified as the primary biological function of hnRNPK. KEGG pathway analysis confirmed that the differential genes were mainly enriched in metabolism signaling pathway.

HnRNPK was a transcription factor among hnRNPA1, hnRNPK, hnRNPR, and hnRNPU, so we speculated that hnRNPK was the core component in the hnRNPK/A1/R/U complex. To identify the biological functions of hnRNPK in colorectal adenocarcinoma cells, the primary biological processes that hnRNPK is involved in were analyzed by RNA-seq in HCT116 cells. The heatmap revealed that 3514 and 3321 genes (upregulated and downregulated, respectively) were differentially expressed after the hnRNPK knockdown (**Figure 7A**). By GO analysis of these differential genes, regulation of transcription by RNA polymerase II was identified as the primary biological function of hnRNPK (**Figure 7B**). In addition, hnRNPK was also involved in regulation of cell cycle, phosphorylation and protein transport. Next, gene set enrichment analysis (GSEA) was also analyzed. Similarly, the regulation of transcription by RNA polymerase II and translation by ribosome were identified as the primary biological processes (**Figures 7C** **, D**). Altogether, our results showed that hnRNPK from the hnRNPK/A1/R/U complex mainly regulated cellular transcription and translation. In FHC cells, the primary biological functions of hnRNPK were further analyzed by RNA-seq. By GO analysis of the differential genes, regulation of DNA binding was identified as the primary biological function of hnRNPK. KEGG pathway analysis confirmed that the differential genes were mainly enriched in metabolism signaling pathway (**Figures 7E** **, F**). Therefore, the hnRNPK-mediated transcription and translation processes are specific to cancer cells but not to the normal cells.

### HnRNPK was a favorable factor in human colorectal adenocarcinoma, promoting immune cell infiltration and inhibiting tumor growth

**Figure 8:**
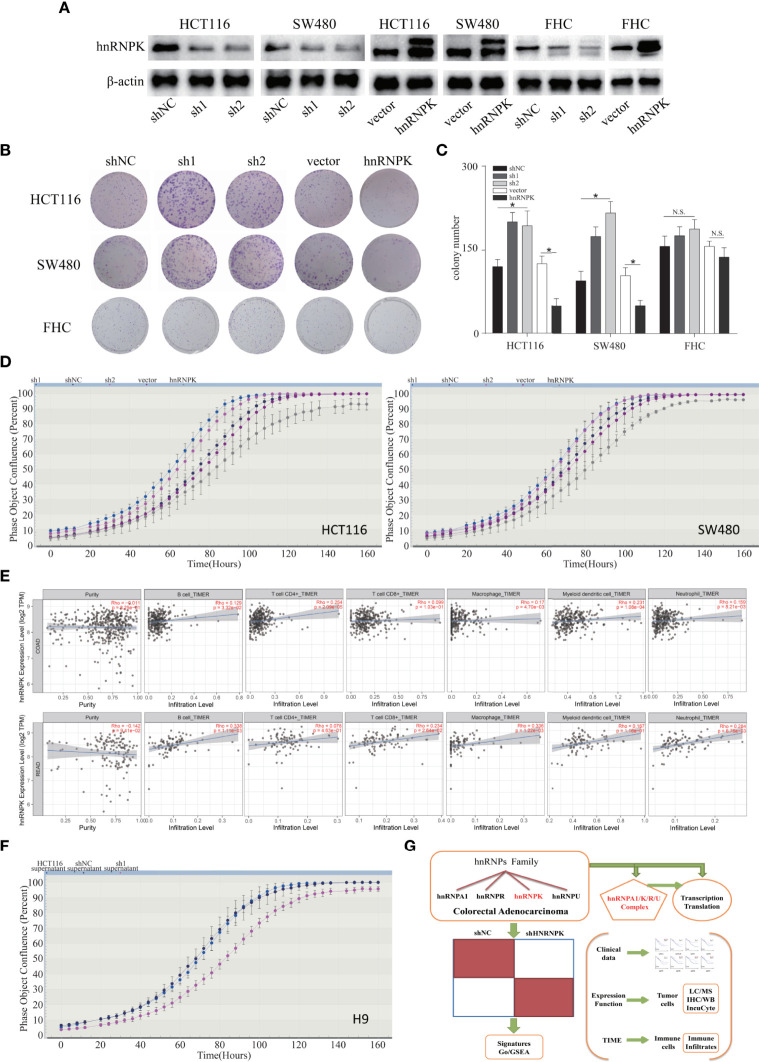
HnRNPK was a favorable factor in human colorectal adenocarcinoma, promoting immune cell infiltration and inhibiting tumor growth. **(A)** Stable hnRNPK-knockdown and hnRNPK-overexpressed cell lines (HCT116, SW480, and FHC) were established by lentiviral infection. WB revealed that the expression of hnRNPK was downregulated or upregulated in a stable manner. **(B, C)** In HCT116 and SW480 cells, the data showed that the cell clonogenicity was promoted after hnRNPK knockdown and attenuated after hnRNPK overexpression. In FHC cells, we found that hnRNPK knockdown or overexpression did not affect the cell clonogenicity. **(D)** IncuCyte-based analysis was used to accurately monitor the cell proliferation. The data revealed that cell proliferation was increased after hnRNPK knockdown and attenuated after hnRNPK overexpression. **(E)** The relationship between hnRNPK expression and infiltration of six immune cell types was analyzed to estimate the effect of hnRNPK on the colorectal adenocarcinoma microenvironment. A significantly positive correlation was observed between hnRNPK overexpression and infiltration of the B cells, CD4+ T cells, macrophages and neutrophil cells. **(F)** After treatment with supernatant of hnRNPK-knockdown cells, the cell proliferation of H9 was inhibited. **(G)** Proposed model for the role of hnRNPK/A1/R/U complex in human colorectal adenocarcinoma. *P < 0.05.

To study the function of hnRNPK *in vitro*, we established stable hnRNPK-knockdown and hnRNPK-overexpressed cell lines (HCT116, SW480, and FHC) by lentiviral infection. WB revealed that the expression of hnRNPK was downregulated or upregulated in a stable manner (**Figure 8A**). For hnRNPK overexpression detection, the upper band showed the overexpression of hnRNPK and the lower band showed its basic expression. We then validated the effect of hnRNPK on cell proliferation and clonogenicity. In HCT116 and SW480 cells, the data showed that the cell clonogenicity was promoted after hnRNPK knockdown and attenuated after hnRNPK overexpression. In FHC cells, we found that hnRNPK knockdown or overexpression did not affect the cell clonogenicity (**Figures 8B, C**). Therefore, the hnRNPK-regulated tumor growth is specific to cancer cells but not to the normal cells. Furthermore, IncuCyte-based analysis was used to accurately monitor the cell proliferation. The data revealed that cell proliferation was also increased after hnRNPK knockdown and attenuated after hnRNPK overexpression (**Figure 8D**). The relationship between hnRNPK expression and infiltration of six immune cell types was analyzed to estimate the effect of hnRNPK on the colorectal adenocarcinoma microenvironment. A significantly positive correlation was observed between hnRNPK overexpression and infiltration of the B cells, CD4^+^ T cells, macrophages and neutrophil cells (*P* < 0.05) (**Figure 8E**). Next, T-cell lymphoma cell line (H9) was used to preliminarily investigate the interaction between cancer cells and T cells. After treatment with the supernatant of hnRNPK knockdown cells, cell proliferation of H9 was significantly inhibited (**Figure 8F**). Our results suggested that hnRNPK was a favorable factor against human colorectal adenocarcinoma, promoting immune cell infiltration and inhibiting tumor growth.

## Discussion

Abnormal gene expressions of hnRNP family have been observed in multiple tumor types (6–8, 35). Recently, the roles of hnRNPs in the occurrence and progression of some tumors have been reported (36–40), but informatic analysis of hnRNPs has not been accomplished. This work is the first to analyze the diverse expression patterns and prognostic values of the most important hnRNPs members, and to validate their expressions in colorectal adenocarcinoma. The aim of this study is to improve treatment for patients with colorectal adenocarcinoma.

Among the hnRNPs, most studies were focused on hnRNPA1 in tumors. For instance, hnRNPA1 was highly expressed in oral squamous carcinoma cells, and this overexpression controlled the alternative splicing of CDK2 exon 5, affecting the regulation of the G2/M cell cycle phase and cell proliferation (41). Moreover, hnRNPA1 promoted cell invasion by inducing EMT transition and was a promising molecular target in gastric cancer cells (42). In colorectal adenocarcinoma, hnRNPA1 was identified as a potential biomarker and contributed to tumor growth by enhancing aerobic glycolysis (43, 44). In this study, we illustrated that the expression of hnRNPA1 was elevated in colorectal adenocarcinoma tissues, which was in line with the previous findings (45). Moreover, mRNA amplification and DNA methylation levels of hnRNPA1 were increased in tumor tissues, indicating that hnRNPA1 overexpression was closely related to the progression of cancers. However, high expression of hnRNPA1 predicted better OS rates in colorectal adenocarcinoma patients, suggesting that hnRNPA1 might be an antitumor factor in tumorigenesis. Therefore, it seems that the function of hnRNPA1 is a double-edged sword, and requires more attention in future studies.

HnRNPA2B1/C/D/F activations are oncogenic events in some human cancers. HnRNPA2B1 interacted and regulated oncogenic KRAS signaling in pancreatic ductal adenocarcinoma cells (40). Additionally, hnRNPC and hnRNPF enhanced cell survival and EMT in breast cancer and bladder cancer by controlling the stabilization of mRNAs (46, 47), and the detection of hnRNPC suggests a negative prognosis in oral squamous carcinoma patients (39). In our work, we found that the expression levels of hnRNPA2B1/C/D/F were upregulated in colorectal adenocarcinoma tissues. Furthermore, the expression levels were positively correlated with each other, which revealed that the hnRNPA2B1/C/D/F network was involved in colorectal adenocarcinoma carcinogenesis. However, the expression levels were not associated with the clinical outcomes of colorectal adenocarcinoma patients, suggesting that hnRNPA2B1/C/D/F were not distinct prognostic biomarkers in colorectal adenocarcinoma. The analysis of clinical outcomes requires larger sample size which is pivotal to the precision and specificity of research.

Based on the results listed above, the prognostic value and functions of hnRNPK/A1/R/U were further investigated. HnRNPs are known to act as tumor suppressor or oncogene in specific cell type manner irrespective of their expression pattern. Most research has verified hnRNPK as an oncogene (12). In bladder cancer, pancreatic cancer and renal cancer, studies have found that hnRNPK promotes cancer cell proliferation (48–50). Moreover, translocation of hnRNPK into nucleus promotes tumor metastases by up-regulating matrix metalloproteinase (51, 52). Recently, hnRNPK was also validated as a tumor suppressor in gastric and hematological tumors (53). In our study, we found that the expression of hnRNPK was enhanced in colorectal adenocarcinoma tissues, which was identical with the findings of Wang et al. (54). But contrary to the results of a study by Carpenter et al. (55), we found that patients with high levels of hnRNPK had better OS rates. The FHC cells are established from human fetal normal colonic mucosa. Because of their origin from normal fetal colon and their relative resistance to the induction of apoptosis, FHC cells have been considered as a valuable experimental control for various studies. Compared with FHC cells, we showed that high expression of hnRNPK reduced the cancer cell proliferation, so we speculated that the patients with higher hnRNPK levels might have lower cell proliferation rates and stronger immune infiltrations, thus prolonging their survival times. Exploring the proliferation regulation mechanism of hnRNPK in colorectal adenocarcinoma was imperative. In this study, many genetic alterations of hnRNPs occurred, in which mRNA upregulation was predominant. We found that the DNA methylation level of hnRNPK was decreased or increased. The upstream mechanism of the expression of hnRNPK needed to be studied further. In addition, hnRNPK is a transcriptional cofactor of p53 in response to DNA damage (56), but the function of hnRNPK is also reported to be independent of p53 status (57, 58). The relationship between hnRNPK and p53 status in colorectal adenocarcinoma will be conducted in our future research.

HnRNPR was reported as a pro-oncogene in gastric cancer development and promoted the transcription of the c-fos oncogene (13). Our data first demonstrated that high expression of hnRNPR existed in colorectal adenocarcinoma and predicted increased OS rates, indicating that hnRNPR is a prognostic biomarker for human colorectal adenocarcinoma. HnRNPU participates in the regulation of multiple biological processes such as chromatin structure and dynamics, telomere recombination, genomic stability, post-translation and modification (59). In cancer progression, an hnRNPU axis regulated chromatin looping, cell apoptosis, and chemotherapy response (14). But until now, the expression and role of hnRNPU in colorectal adenocarcinoma has been unclear. In the present study, we discovered that the expression of hnRNPU in colorectal adenocarcinoma was increased, while the DNA methylation level of hnRNPU was decreased. DNA methylation is a pivotal epigenetic modification that is involved in control mechanisms of gene expression regulation (60). Owing to the classical regulatory processes happening at gene promoter regions, we hypothesized that certain tumor-specific protein molecules bound specifically to the hnRNPU promoter to downregulate the DNA methylation. Unlike other members, DNA methylation of hnRNPU might be modulated by several complex factors, and the molecular mechanisms should be further explored.

The modifications of hnRNP proteins are frequently focused on post-translational levels. It is still unclear about the transcriptional modifications of hnRNPs. Recently, the role of DNA methylation in controlling the activities of gene promoters, whether CpG islands or non-CpG islands, has been extensively investigated. In mammals, DNA methylation in promoters is well known to suppress gene expression and is the presumed therapeutic target of methylation inhibitors (61). In READ tissues, hnRNPC, hnRNPK, hnRNPF, and hnRNPU had lower promoter methylation levels and higher expression levels, which was consistent with this view. In addition, the transcriptional activity of a gene is also controlled by the packaging of the template within chromatin. Chromatin is the natural substrate for the control of gene expression. Chromatin contains DNA and structural proteins such as histones. DNA methylation and histone modification can alter the nucleosomal infrastructure to repress or activate transcription (62). Therefore, DNA methylation and chromatin are critical for the transcription initiation and gene expression. In this work, hnRNPA1, hnRNPA2B1, hnRNPF, and hnRNPK had higher promoter methylation levels and higher expression levels in COAD tissues. We hypothesized that their higher methylation levels might simultaneously modulate the chromatin structure and make the upstream regulators or activators getting access to the DNA and enhancing their transcription. The transcription of hnRNPs may be a result of the interaction between DNA promoter methylation and chromatin structure. Nevertheless, we have no evidences for the possible regulatory relationship between expressions and DNA methylation levels of hnRNPs. The current databases are unable to analyze and provide the correlation data. The potential regulatory molecular mechanisms will be investigated in our future studies.

Cancer cells require high levels of transcription to survive and maintain their cancerous phenotype. In the tumorigenesis of colorectal adenocarcinoma, chromosome instability, hypoxia and transcription inhibitors used in the treatment of cancer may generate a stress situation in which the cancer cell responds by overexpressing hundreds of genes (63). The levels of hnRNPK/A1/R/U were significantly correlated with and associated with better OS rates, while the levels of hnRNPs were increased. These seemingly contradictory results may be a stress response, causing the up-regulation of hnRNPK/A1/R/U. Immune response is the frontline of defense against cancer. A dynamic and coordinated gene expression program lies at the heart of the immune response. Alternative splicing and mRNA stability have been shown to be regulated during the immune response and participate in modulating gene expression in a specific manner (64, 65). HnRNPs are nucleic acid-binding proteins which mainly regulate alternative splicing and mRNA stability. In this work, we observed an obvious correlation between hnRNPK overexpression and immune infiltrations. Therefore, we hypothesized that the increased levels of hnRNPs might be emerging as cascade response of the immune process. The possible evidences will be explored in the future. We also identified and validated the existence of hnRNPK/A1/R/U complex by proteomic technology. In our results, many transcription and translation genes were identified to be associated with the hnRNPK/A1/R/U network. Gene expression is generally dependent on protein synthesis, in which ribosomes recognize and decode mRNA templates *via* translation initiation, extension, and termination. The ribosome is a complex organelle composed of many different proteins and nucleic acids, and is responsible for protein synthesis in living cells (66). Moreover, the poly(A)-binding process plays pivotal roles in the translation and stability of the mRNA (67). Therefore, we speculated that the hnRNPK/A1/R/U network processed the transport and stabilization of mRNAs in the cytoplasm, and modulated mRNA translation in the ribosome. The GO and KEGG analysis results validated that translation and transcription were the predominant processes regulated by the network.

HnRNPK was the only transcription factor among hnRNPA1, hnRNPK, hnRNPR, and hnRNPU. Moreover, from the survival analysis results in HPA and GEPIA webtools, only hnRNPK was associated with better OS rates for colorectal adenocarcinoma patients. Therefore, we hypothesized that hnRNPK might be the core component in the hnRNPK/A1/R/U complex. The GO and GSEA analysis data by RNA-seq validated that hnRNPK mainly regulated cellular transcription and translation, which was identical to the function of hnRNPK/A1/R/U network. The above results indicated that hnRNPK was a primary regulatory factor for development of colorectal adenocarcinoma. *In vitro*, we preliminarily investigated the role of hnRNPK in colorectal adenocarcinoma. The data showed that a positive correlation was observed between hnRNPK overexpression and infiltration levels of B cells, CD4+ T cells, macrophages and neutrophil cells. Hence, we hypothesized that the overall immune infiltration levels of hnRNPK/A1/R/U complex might be enhanced. T-cell lymphoma cell line (H9) was used to investigate the interaction between cancer cells and T cells. After treatment with the supernatant of hnRNPK-knockdown cells, cell proliferation of H9 was significantly inhibited. Through proliferation test, we preliminarily identified that hnRNPK was a favorable factor against human colorectal adenocarcinoma and promoted immune infiltration, thus inhibiting tumor growth. Notably, the biological functions and molecular mechanisms of hnRNPK/A1/R/U complex in colorectal adenocarcinoma still need future investigations.

## Conclusion

As summarized in **Figure 8G**, we systematically evaluated the expression profiles and prognostic values of the eight most important subtypes of hnRNPs in colorectal adenocarcinoma, and presented thorough findings of the potential molecular biological characteristics of hnRNPs. Our results illustrated that the elevated levels of hnRNPK/A1/R/U in colorectal adenocarcinoma tissues exert critical roles in the tumorigenesis of colorectal adenocarcinoma. Importantly, we validated that the hnRNPK/A1/R/U complex is a favorable prognostic biomarker of colorectal adenocarcinoma. Targeting transcription factor hnRNPK during transcription and translation could be a promising therapeutic potential for colorectal adenocarcinoma treatment.

## Data Availability Statement

The datasets presented in this study can be found in online repositories. The names of the repository/repositories and accession number(s) can be found in the article/supplementary material.

## Ethics Statement

The studies involving human participants were reviewed and approved by Ethics Committee of The First Affiliated Hospital of Zhengzhou University. The patients/participants provided their written informed consent to participate in this study.

## Author Contributions

ZG and YQ conceived and designed the study. YL obtained information from the informatic tools. YL, HW, QM, and JW performed the experiments and acquired the result data. ZG and YL drafted the manuscript. ZG and QM critically revised the manuscript and supervised the study. All authors contributed to the article and approved the submitted version.

## Funding

This work was supported by the funds from the National Natural Science Foundation of China (Grant No. 81902833, 82103113), the Henan Provincial Science and Technology Research Project (Grant No. 212102310112, LHGJ20210286) and the Certificate of Postdoctoral Research Grant of Henan Province.

## Conflict of Interest

The authors declare that the research was conducted in the absence of any commercial or financial relationships that could be construed as a potential conflict of interest.

## Publisher’s Note

All claims expressed in this article are solely those of the authors and do not necessarily represent those of their affiliated organizations, or those of the publisher, the editors and the reviewers. Any product that may be evaluated in this article, or claim that may be made by its manufacturer, is not guaranteed or endorsed by the publisher.
